# Development of a New Voltammetric Method for Aceclofenac Determination on Glassy Carbon Electrode Modified with Hierarchical Nanocomposite

**DOI:** 10.3390/s22228954

**Published:** 2022-11-18

**Authors:** Anna Górska, Beata Paczosa-Bator, Justina Gaidukevič, Robert Piech

**Affiliations:** 1 Faculty of Materials Science and Ceramics, AGH University of Science and Technology, al. Mickiewicza 30, 30-059 Krakow, Poland; 2 Faculty of Chemistry and Geosciences, Vilnius University, Naugarduko str. 24, LT-03225 Vilnius, Lithuania

**Keywords:** aceclofenac, metal nanoparticles, voltammetry, carbon nanomaterials

## Abstract

Aceclofenac (ACL) is an anti-inflammatory drug, which is taken by patients who mainly suffer from rheumatoid conditions. In this work, we propose a new voltammetric method that allows the determination of ACL in pharmaceutics, urine, and plasma. As a working electrode, a glassy carbon electrode (GCE) modified with carbon nanofibers, carbon nanotubes, and NiCo nanoparticles (eCNF/CNT/NiCo-GCE) was used. The mentioned sensors are characterized by good repeatability and sensitivity, and their process of preparation is simple, fast, and cost-effective. Instrumental and method parameters were optimized, and the influence of interferences was investigated. To validate the analytical performance of the method, calibration was conducted. Good linearity was obtained (0.05–1.4 µM, r = 0.998), as well as excellent limit of detection (LOD) and limit of quantification (LOQ) values (0.7 nM and 2.1 nM, respectively). Calculated recoveries that were in the range of 98%–105% indicate that this method is accurate and might be used in routine laboratory practice.

## 1. Introduction

**Figure 1:**
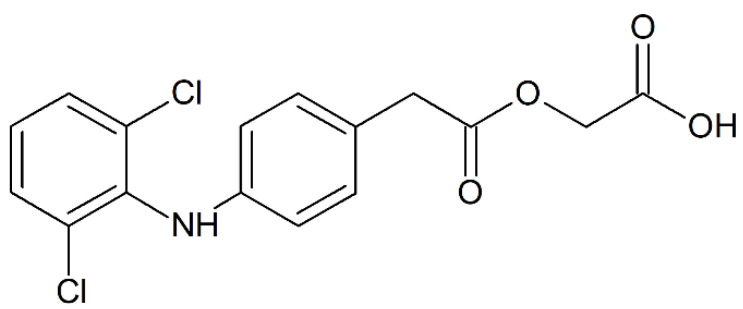
Structural formula of ACL.

Aceclofenac (Figure 1) belongs to the group of medications called nonsteroidal anti-inflammatory drugs (NSAID). In addition to its anti-inflammatory properties, ACL can also provide pain relief. It is used in the therapy of patients suffering from ankylosing spondylitis, rheumatoid arthritis, and osteoarthritis [1,2,3]. The literature reports different analytical methods that might be used for ACL determination. Among them are, for example, high-performance liquid chromatography (HPLC) [4,5,6], spectrophotometry [7,8], spectrofluorimetry [8], and capillary electrophoresis [9]. The most common are chromatographic methods. Researchers use them for ACL determination in pharmaceuticals products and plasma. The lowest value of LOD was achieved using ultra-performance liquid chromatography, and was equal to 0.01 ng mL^−1^. Another popular method is spectrophotometry, which is used mainly for measurements of pharmaceutical products. The lowest LOD achieved by the researchers was equal to 0.20 µg mL^−1^. Capillary electrophoresis was used for ACL determination in urine and wastewater, and the LOD for this method was equal to 0.86 ng mL^−1^. Voltammetry can also be employed for ACL determination [10,11,12,13]. Compared to other methods, voltammetry is characterized by a relatively low cost of analysis, high sensitivity, the ability to obtain low detection limits, and low consumption of chemicals. This electrochemical method has a wide range of applications and can be used to determine organic [14,15] and inorganic [16,17] substances. The most important part of each voltammetric system is a working electrode (WE). The group of sensors that are becoming more and more popular nowadays are solid electrodes, among them, the glassy carbon electrode (GCE) and the carbon paste electrode (CPE). They are characterized by a wide range of working potential, good chemical and mechanical resistance, eco-friendly character, and the most important possibility of modification. Surface modification is something that allows one to improve parameters such as sensitivity, selectivity, and catalytic properties. There are various groups of substances that are used as surface modifiers, e.g., carbon materials (e.g., nanotubes, graphene, carbon black) [18,19], metals and metal oxides (e.g., Pt, Au, TiO_2_, RuO_2_) [20,21], and polymers (e.g., polyaniline, chitosan, PEDOT) [22,23]. Another interesting group is hybrid materials [24,25,26,27], which are the combination of two or more components. It allows a material to be obtained with brand new properties in comparison with the components themselves. Therefore, the current trend in the modification of solid electrodes is mainly focused on this type of material.

There are only a few reports about voltammetric methods designed for the determination of ACL. The lowest value of LOD (2.6 nM) was obtained by researchers who were using multiwalled carbon nanotubes-modified pencil graphite electrode [13]. The sensor was successfully applied to determine ACL in pharmaceutical and urine samples. Other constructions that have also been described in the literature are modified CPE [10,11] and another graphite electrode-based sensor [12].

In this work, we present a voltammetric sensor based on GCE modified with a hierarchical nanocomposite made of carbon nanofibers, carbon nanotubes, and NiCo nanoparticles (eCNF/CNT/NiCo-GCE). The developed sensor is characterized by desirable properties, e.g., good electrical conductivity, electrocatalytic properties, stability, and repeatability in terms of its preparation. The modification of GCE has been performed using the drop-casting method, which is simple, fast, and cost-effective. The described sensor was used in the determination of ACL in pharmaceutical products, urine, and plasma. To the best of our knowledge, there are no reports about voltammetric sensors based on such nanostructures, despite our previously published work [28]. 

The main novelty of the work is the application of eCNF/CNT/NiCo modifier for the modification of GCE and its utilization in aceclofenac determination. To the best of our knowledge, this is the first time GCE has been used in this application. Additionally, our solution presents the best results concerning LOD and LOQ values in comparison with other voltammetric methods for ACL determination. 

## 2. Experimental

### 2.1. Apparatus

For all voltammetric measurements, the multipurpose electrochemical analyzer M161 and the electrochemical stand M164 (both mtm-anko, Kraków, Poland) were utilized. A typical three-electrode system consisted of eCNF/CNT/NiCo-GCE as the working electrode, Ag/AgCl in 3 M KCl as the reference electrode, and platinum wire as the auxiliary electrode. The electrodes were immersed in a quartz cell (total volume 20 mL) filled up with 0.025 M HClO_4_ (10 mL). The electrolyte in the cell was stirred using a Teflon stir bar and a magnetic stirrer (~500 rpm). All pH measurements were conducted using a multifunction meter (Elmetron CX-705, Zabrze, Poland). The dispersion and solvents were sonicated using an ultrasonic bath (Intersonic IS-1K, Olsztyn, Poland) or ultrasonic processor (Vibra-cell VCX 130, Newtown, CT, USA).

### 2.2. Chemicals and Glassware

All chemicals used were of analytical grade and were used without further purification. The standard solution of ACL (0.01 M) was prepared based on ACL reference material (98%, LGC Standard, Wesel, Germany) by dissolution in DMSO (p.a., 99.7%, POCH, Gliwice, Poland). Solutions with lower concentrations were prepared by a suitable dilution of standard with DMSO. All standards were kept refrigerated at 4 °C. Other chemicals were purchased as follows: lyophilizate urine, Medidrug^®^ Basis-line U—Medichem, Germany; human plasma—Biowest, France; dimethylformamide, DMF (p.a.), methanol (p.a.), boric acid (p.a.), sodium hydroxide (p.a.)—POCH, Poland; Al_2_O_3_ –> 99%, Buehler Micropolish, Lake Bluff, IL, USA; perchloric acid (Suprapur, 70%), trichloroacetic acid (TCA) (p.a.), phosphoric acid (p.a., ≥85%), acetic acid (p.a., ≥99.8%)—Sigma Aldrich, Darmstadt, Germany. Before use, glassware was cleaned using HNO_3_ solution and double-distilled water. All water solvents were prepared using double-distilled water. 

### 2.3. Sample Preparation

#### 2.3.1. Tablets

According to the producer’s declaration, the sample in the form of a tablet contains 100 mg of ACL. Before measurement, 3 tablets were weighed and then crushed in an agate mortar. The appropriate amount of the powder sample was weighed, transferred to the beaker (25 mL volume), dissolved in 10 mL of DMSO, and sonicated for 10 min. In the next step, the sample was filtrated using a cellulose syringe filter (0.45 µm pore size) and the filtrate obtained was transferred to the volumetric flask (25 mL volume) and filled up to the mark with DMSO. 

#### 2.3.2. Lyophilizate Urine

Directly before measurement, lyophilizate urine (Medidrug^®^ Basis-line U) in a vial was dissolved in 5 mL of double-distilled water as suggested by a producer. After that, the sample was filtrated using a cellulose syringe filter (0.2 µm pore size) and appropriately diluted with the supporting electrolyte solution. 

#### 2.3.3. Plasma

The plasma sample purchased from Biowest was stored in a freezer at the temperature −20 °C (as suggested by a manufacturer) and defrosted directly before measurement. In the next step, 800 µL of serum was mixed with 200 µL of 10% TCA and put in the laboratory shaker for 5 min and then centrifuged for 30 min. The supernatant was collected, filtrated using a cellulose syringe filter (0.2 µm pore size), and appropriately diluted with the supporting electrolyte solution. 

### 2.4. Fabrication of the eCNF/CNT/NiCo-GCE Sensor

#### 2.4.1. Preparation of the Nanocomposite

##### Electrospinning

In the first step, precursor nanofibers were synthesized using the electrospinning method. The spun solution consisted of a mixture of pure PAN and PAN-terpolymer, Ni(Acac)_2_, and Co(Acac)_2_ in DMF. The parameters of the electrospinning process were as follows: applied voltage—9 kV; nozzle diameter—1.1 mm; nozzle-collector distance—40 mm; temperature—30 °C; humidity in the chamber—approximately 15%; duration—30 min.

##### Thermal Treatment

**Figure 2:**
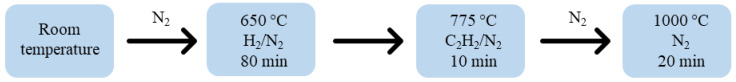
A scheme presenting the conditions of each step of the thermal treatment of nonwovens in a tubular furnace.

Nonwovens prepared by electrospinning underwent the thermal stabilization treatment in the atmosphere of air. The material was annealed, first at 240 °C (for 30 min) and then at 260 °C (for 20 min). It allows the carbon yield in the final material to be increased, and the metal acetylacetonates to be decomposed into metal oxides. In the next step, the nonwovens were subjected to a series of thermal treatments in the tubular reactor, according to the scheme presented in Figure 2. Such a procedure ensured the reduction of metal oxides into metal nanoparticles, the growth of carbon nanotubes, and the full carbonization of nanofibers. Other details related to the synthesis of the eCNF/CNT/NiCo were fully explained in previously published work [28]. Additionally, the results of the SEM, HRTEM, XRD, and Raman spectroscopy characterization of the nanocomposite are also available in the mentioned paper.

The prepared nanocomposite was crushed in the mortar and mixed with DMF to obtain a suspension with a concentration of 1 mg mL^−1^. To ensure good homogenization, the suspension was sonicated for 10 min using an ultrasonic processor (Vibra-cell VCX 130), and after that was used for GCE modification. 

#### 2.4.2. Modification Process

The working electrode was prepared by modification of GCE with the eCNF/CNT/NiCo dispersion by a drop-casting method. GCE was chosen for this purpose due to the simplicity of the process of its modification. Additionally, GCE is characterized by a wide working potential range and good chemical and mechanical properties.

In the first step, the surface of GCE was polished on Al_2_O_3_ slurry (particle size 0.3 µm) in order to remove adsorbed contamination and to smooth the surface. Then, GCE was rinsed in the stream of double-distilled water to remove polishing powder and placed in the methanol–water mixture and sonicated for 3 min. After that, the GCE was dried and ready to be used. Before use, the eCNF/CNT/NiCo modifier dispersion was sonicated for 15 min to avoid agglomeration of the particles and applied on the surface of the GCE by drop-casting method. The modified GCE should dry for approximately 2 h to ensure evaporation of DMF. 

### 2.5. Measurement Procedure

Quantitative measurements of ACL were carried out using the differential pulse voltammetry (DPV) technique. The supporting electrolyte consisted of 0.025 M HClO_4_. Measurements were conducted according to the following procedure:

- Cleaning of the GCE surface: E = 1102 mV; t = 3 s;
- Accumulation: E_acc_ = 500 mV; t_acc_ = 20 s;
- Rest period: 3 s;
- Registration of ACL voltammograms in the potential range: 500–1100 mV.

Other parameters of the DPV technique were set as follows: t_s_ = 10 ms; t_w_ = 5 ms; E_s_ = 6 mV; ΔE = 100 mV. 

## 3. Results

### 3.1. Sensor Characterization

#### 3.1.1. Volume of Modifier 

**Figure 3:**
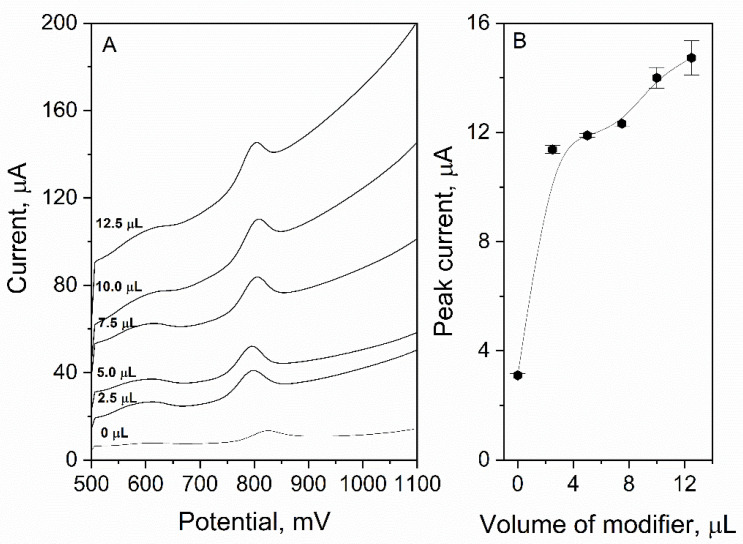
The dependence of ACL peak current on volume of eCNF/CNT/NiCo modifier applied on the surface of GCE, DP voltammograms (**A**), plot (**B**). The supporting electrolyte consisted of 0.025 M HClO_4_, and the ACL concentration was equal to 3 µA, E_acc_ = 500 mV, t_acc_ = 20 s.

In order to obtain the highest sensitivity and maintain stability of the register signal, optimization of the volume of the surface modifier that is applied on GCE should be performed. In the experiment, six GCEs were prepared and then modified with the following volume of eCNF/CNT/NiCo dispersion: 0, 2.5, 5.0, 7.5, 10.0, 12.5 µL. Each electrode was used to measure the signal of 3 µM of ACL (in 0.025 M HClO_4_). The results presented in Figure 3 show that the ACL peak current increased with the volume of the modifier (for 0 µL: I_p_ = 3.10 ± 0.06 µA; for 12.5 µL: I_p_ = 14.7 ± 0.6 µA). However, it is worth mentioning that for 10.0 and 12.5 µL, the signal stability was poorer than it was for lower volumes (error bars in Figure 3B). For 7.5 µL, signal stability was good (RSD = 0.9%) and the ACL peak current was relatively high (I_p_ = 12.3 ± 0.1 µA); therefore, this value was chosen as optimal for future measurements. Additionally, it can be observed that the peak potential shifted toward less positive values with an increasing modifier volume applied on the electrode. It proves that eCNF/CNT/NiCo nanocomposite exhibits catalytic properties. 

#### 3.1.2. Repeatability and Reproducibility 

To investigate the reproducibility of the electrode preparation, an appropriate experiment was conducted. For this purpose, five GCEs were modified with 7.5 µL of eCNF/CNT/NiCo dispersion and used for ACL measurement. For each sensor, the signal was registered nine times and then the average peak current value was estimated. The RSD% calculated based on the results obtained was equal to 6.2% (n = 5, ACL concentration 3 µM). It is a good result and shows the proper reproducibility of the sensor preparation. The repeatability of the signal measured on eCNF/CNT/NiCo-GCE was tested using one sensor from the experiment mentioned above. For that purpose, the signal from 0.1 µM ACL was measured. Based on the obtained voltammograms, the RSD% value was calculated and was equal to 1.2% (n = 9). Such a result suggests that the repeatability of the signal measured on the developed sensor is satisfactory. 

#### 3.1.3. Stability of Sensor

To estimate the stability of work, we counted the number of measurements that could be performed using one sensor without deterioration of its sensitivity. The experiment was conducted in 0.025 M HClO_4_ for an ACL concentration equal to 3 µM. The results showed that eCNF/CNT/NiCo-GCE can perform at least 600 measurements. 

### 3.2. Aceclofenac Behavior on the Surface of eCNF/CNT/NiCo-GCE

**Figure 4:**
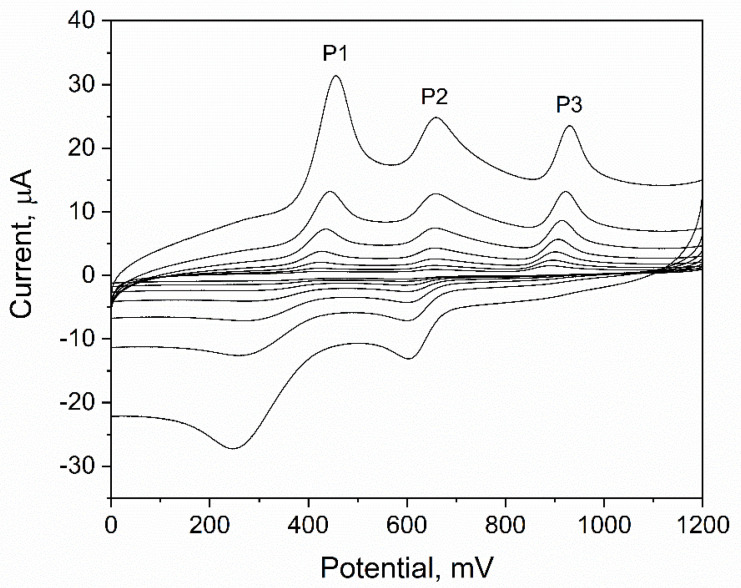
LSV CV measurement of 50 µM ACL in 0.025 M HClO_4_ for different scan rates from the range 6.3–500 mV s^−1^.

The electrochemical behavior of ACL on the surface of eCNF/CNT/NiCo-GCE was investigated using cycling voltammetry (CV) measurements and the linear sweep voltammetry (LSV) technique. Measurement was carried out in 0.025 M HClO_4_ for an ACL concentration equal to 50 µM. During the experiment, scan rate values were changed from 0.0063 V s^−1^ to 0.5 V s^−1^. The results obtained (Figure 4) revealed the occurrence of three oxidation and two reduction peaks. During quantitative measurements with the DPV technique, more than one oxidation peak was also observed, which is consistent with the LSV results. However, the results showed that only the peak marked as P3 was analytical (linear current response when the analyte concentration was changed). 

#### The Mechanism of the Electrochemical Reaction

The occurrence of three oxidation and two reduction peaks suggests that the electrochemical reaction of ACL is a three-step process, where two steps are reversible. To check whether the nature of each step is diffusion or adsorptive, the dependence of the natural logarithm of the peak current on the natural logarithm of the scan rate for each oxidation peak was plotted, and then the slope value of each equation was analyzed. The following results were obtained: P1: lnI_p1_ = 1.03 ln*v* − 3.45; r = 0.999—adsorption, P2: lnI_p2_ = 0.71 ln*v* − 2.27; r = 0.999—mixed character adsorption/diffusion, P3: lnI_p3_ = 0.58 ln*v* − 1.01; r = 0.993—diffusion. As might be observed, the character of each reaction was different. In the next step, calculations that allow us to estimate the number of electrons taking part in the electrochemical reaction were made. For this purpose, the Tafel plot was made based on data obtained from the rising part of the LSV voltammogram registered for a scan rate equal to 0.0125 V s^−1^. The results obtained allowed us to determine the slope (8.15 dec V^−1^), which was later used to calculate the transfer coefficient, α from the Tafel equation (slope = (1 − *α*)*Fn*/2.3*RT*). The calculated value of *α* was equal to 0.76. In further steps, the number of electrons, *n*, that participated in the electrochemical reaction was calculated. The Laviron equation for processes controlled by diffusion was used (slope = *RT*/2*Fαn*). The slope was taken from the dependence of the P3 peak potential on the natural logarithm of the scan rate, and *α* was taken from the calculations of the Tafel plot. Calculations revealed that n = 1.78, which suggests that the number of electrons taking part in the electrochemical reaction is 2. 

**Figure 5:**
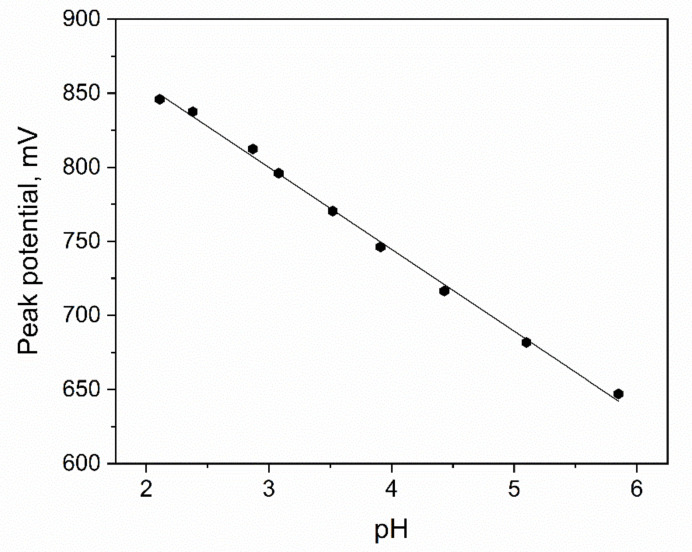
Dependence of the ACL peak potential on pH of Britton–Robinson buffer.

**Figure 6:**
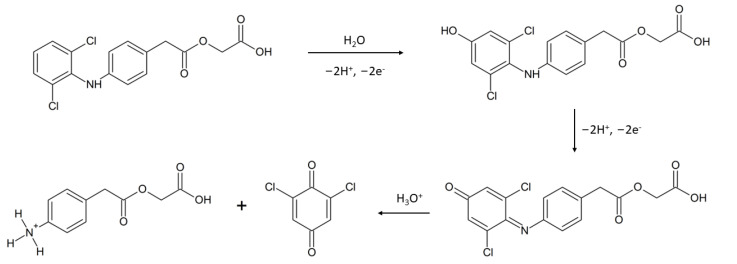
Proposed mechanism of ACL electrochemical reaction [10,11,13].

To bring closer the possible mechanism of the ACL electrode reaction, the dependence of the ACL peak potential on the pH of the supporting electrolyte was plotted. Data were collected from the experiment carried out using Britton–Robinson buffer with a pH in the range of 2.1 to 5.9. The concentration of ACL was equal to 3 µM, and the accumulation potential and time were equal to 500 mV and 20 s, respectively. In the tested pH range, a linear dependence was obtained (Figure 5 and Equation (1)) with a slope equal to −55.3 mV pH^−1^. This result is close to the theoretical value and means that an equal number of protons and electrons were exchanged in the electrode process. On the basis of the collected data, it was concluded that the ACL electrode reaction involved two protons and two electrons. The results obtained are consistent with the literature and confirm the mechanism already proposed in previous papers (Figure 6) [10,11,13].

(1) $$E_{p}=-55.3\;pH+965.9\;mV\;\;\;\;\;\;\;\;\;\;\;\;r=0.999$$

### 3.3. Supporting Electrolyte Optimization

#### 3.3.1. The Type of Supporting Electrolyte

The choice of supporting electrolyte is an important part of optimalization during the development of the voltammetric method. It has an influence on both the peak current and its potential. During the experiment, the accumulation potential and time were equal to 500 mV and 20 s, respectively, and the ACL concentration was 3 µM. The influence of the following electrolytes on the ACL signal was investigated: HClO_4_, H_3_PO_4_, HCl, CH_3_COOH, acetate buffer pH 4.5, phosphate buffer pH 7.0, borate buffer pH 9.1 (concentration of each electrolyte 0.025 M). In the case of phosphate and borate buffers, no signal of ACL was observed. An ACL peak was observed in the remaining electrolytes: HClO_4_: I_p_ = 10.87 ± 0.05 µA, E_p_ = 827 mV; H_3_PO_4_: Ip = 7.2 ± 0.1 µA, E_p_ = 861 mV; HCl: I_p_ = 7.3 ± 0.2 µA, E_p_ = 858 mV; CH_3_COOH: I_p_ = 0.27 ± 0.02 µA, E_p_ = 818 mV; acetate buffer: I_p_ = 0.57 ± 0.01 µA, E_p_ = 723 mV. The results show that the highest signal was obtained in HClO_4_. Additionally, the mentioned electrolyte provided a stable signal and a favorable peak-to-background ratio; therefore, it was chosen for further measurements. 

#### 3.3.2. The Concentration of Supporting Electrolyte

The optimization of concentration of the supporting electrolyte was performed in conditions analogous to those in the previous experiment. The influence of HClO_4_ concentration was investigated in the range from 0.025 M to 0.2 M. The results showed that the lower the concentration, the higher the ACL peak current. For 0.025 M, the peak current was 11.23 ± 0.09 µA and additionally, background current was low; therefore, this value was chosen as optimal for further voltammetric measurements. It is also worth mentioning that HClO_4_ is a strong oxidizing agent—its high concentrations could damage the modifier layer or decompose the measured analyte. This is another reason why using a low concentration of the chosen supporting electrolyte is beneficial. 

### 3.4. DPV Parameters Optimization

The parameters of the DPV technique strongly influence the sensitivity of the voltammetric method. Therefore, their optimization is an important part of the research. The conditions of the experiment were as follows: the electrolyte consisted of 0.025 M HClO_4_, the accumulation potential and time were 500 mV and 20 s, respectively, and the ACL concentration was equal to 3 µM. The first optimized parameters were sampling time, t_s_, and waiting time, t_w_. Both of them were tested in the range from 5 to 40 ms. The results showed that the highest ACL peak current and a well-shaped peak were obtained for a t_s_ and t_w_ equal to 10 ms and 5 ms, respectively (pulse time 15 ms); therefore, these values were used in further measurements. It means that a time equal to 5 ms (t_w_) is sufficient to reduce the capacitive current and that 10 ms (t_s_) is necessary to measure the faradaic current associated with the electrochemical reaction of the analyte. The influence of the step potential, E_s_, was tested in the range of 1–6 mV (higher values caused deterioration of the current signal). This parameter has an influence on the rate of change of the electrode potential over time. Measurements showed that the higher the E_s_, the higher the ACL peak current (due to an increase of the scan rate); therefore, 6 mV was chosen as optimal. The influence of the pulse amplitude, ΔE, was investigated in the following range: 5–100 mV (positive and negative mode). The higher the parameter value, the higher the ACL peak current; therefore, ΔE = 100 mV was chosen as optimal. Despite the fact that the current signal was increasing with the ΔE, higher values of the parameter were not tested due to deterioration of the signal. 

### 3.5. Accumulation Potential and Time Optimization 

**Figure 7:**
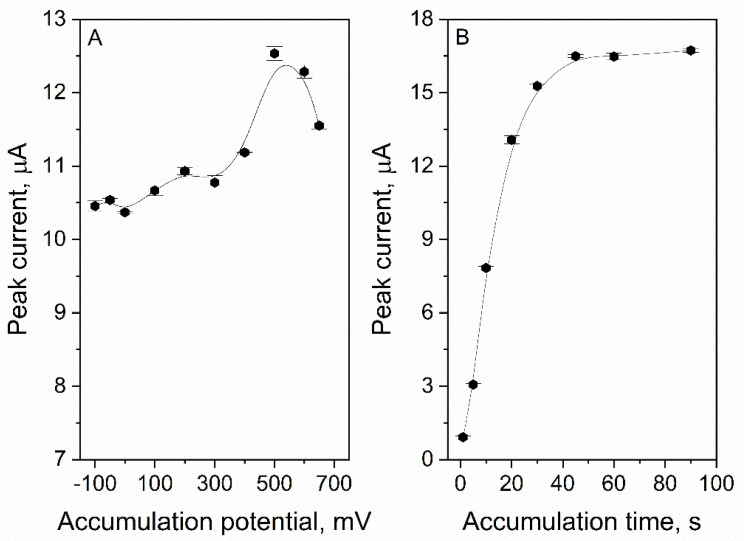
The dependence of ACL peak current on accumulation potential (**A**) and time (**B**). Supporting electrolyte: 0.025 M HClO_4_, ACL concentration 3 µM.

The accumulation potential and time are characteristic parameters of the stripping voltammetry. The use of the mentioned technique allows for an improvement in the sensitivity of the method; therefore, lower concentrations of the analyte can be determined. The conditions of the experiment were the following: the supporting electrolyte consisted of 0.025 M HClO_4_ and an ACL concentration equal to 3 µM. The results presented in Figure 7A clearly show that an accumulation potential equal to 500 mV was optimal for the determination of ACL. For this value, the ACL signal was the highest (12.5 ± 0.1 µA) and the peak is well-shaped. When the accumulation time was extended, the ACL peak current increased (Figure 7B). The maximum peak current was obtained for an accumulation time equal to 45 s (16.50 ± 0.06 µA), and after exceeding this value, no further increase was observed. This effect was probably caused by the coating of the electrode surface with the analyte. Active centers where the catalytic reaction took place are covered, and more ACL particles could not be oxidized; therefore, an increase in the peak current was not observed. For routine analysis, t_acc_ = 20 s could be used—the ACL signal is relatively high and the measurement can be performed quickly. When higher sensitivity is required, a longer time (e.g., 45 s) could be applied. 

### 3.6. Interferences Study

The study of interferences is an important part of the work when a new analytical method is developed. It allows to define how potential components of the sample matrix might affect the measurement and to determine what kind of sample preparation method should be used. During the experiment, the influence of organic and inorganic substances on the ACL signal was investigated. All measurements were conducted in 0.025 M HClO_4_, the accumulation potential and time were equal to 500 mV and 20 s, respectively, and the ACL concentration was equal to 3 µM. The impact of the following substances was tested: Fe (III), Pb (II), Zn (II), Cu (II) (30 µM added), Mg (II), Ca (II) (150 µM added), SO_4_^2−^, NO_3_^−^, Cl^−^, CO_3_^2−^ (1000 µM added), citric acid, ascorbic acid, glucose (500 µM added), uric acid, caffeine, lactose monohydrate, aspartame (100 µM added), magnesium stearate, microcrystalline cellulose (5 mg per 10 mL of electrolyte), and Triton X-100 (15 ppm added). Among all tested substances in the mentioned concentration range, only the occurrence of Fe (III) disturbed the measurement. Fe (III) in a concentration equal to 3 µM caused a 42% change in the peak current value.

### 3.7. Analytical Performance

**Figure 8:**
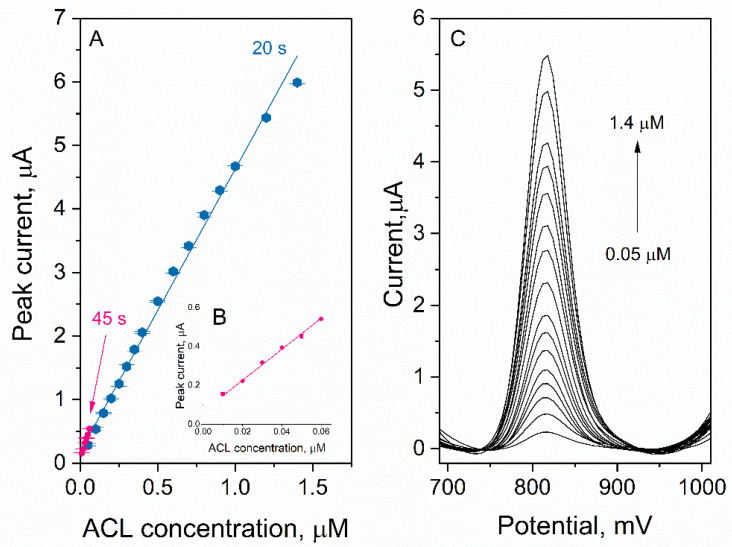
(**A**) Regressions of calibrations carried out for t_acc_ = 20 s (blue line) and t_acc_ = 45 s (pink line), (**B**) Regression for t_acc_ = 45 s in magnification, (**C**) DP voltammograms corresponding to the calibration for t_acc_ = 20 s.

To validate the analytical performance of the developed method for ACL determination, calibration was performed. The experiment was carried out in 0.025 M HClO_4_, and the accumulation potential and the time were equal to 500 mV and 20 s, respectively. A linear response (Figure 8A) was obtained in the concentration range of 0.05–1.4 µM (slope: 4.45 ± 0.07 µA µM^−1^, intercept: 0.18 ± 0.05 µA, r = 0.998). On the basis of the results obtained, LOD and LOQ values were calculated and were equal to 4.7 nM and 14.1 nM, respectively (LOQ = 3 LOD; LOD = 3.3 s/b; where s—signal nosie, b—slope of the calibration). To improve sensitivity and to achieve a lower detection limit, a longer accumulation time could be used. In the next experiment, calibration for t_acc_ = 45 s was carried out. The linear response (Figure 8A,B) was obtained in the concentration range 0.01–0.06 µM (slope: 8.0 ± 0.2 µA µM^−1^, intercept 0.067 ± 0.007 µA, r = 0.999). The calculated LOD and LOQ values were equal to 0.7 nM and 2.1 nM, respectively. A comparison of LOD and LOQ values for ACL determination using different voltammetric methods and the one developed by us was gathered and is shown in Table 1. To validate the applicability of the developed voltammetric method, measurements of real samples were carried out. For this purpose, a pharmaceutical product containing ACL, urine and plasma samples were used. All measurements were carried out using the standard addition method and the procedure described in Section 2.5 (*Measurement procedure*). The samples were prepared as described in Section 2.3 (*Sample preparation*) and diluted appropriately. The results obtained are presented in Table 2. The recoveries were in the range 98%–105%, which suggests that the accuracy of the method is satisfactory. 

## 4. Conclusions

A new highly sensitive voltammetric method for ACL determination was developed. As a working electrode, GCE modified with hierarchical nanocomposite made of carbon nanofibers, carbon nanotubes, and NiCo nanoparticles was used. Measurement conditions such as supporting electrolyte, instrumental parameters, and stripping parameters were optimized. A detailed interference study was also carried out. On the basis of the calibrations obtained (for t_acc_ = 20 s and t_acc_ = 45 s), LOD and LOQ values were calculated. For t_acc_ = 45 s, LOD and LOQ were equal to 0.7 nM and 2.1 nM, respectively. To the best of our knowledge, this is the best result in comparison to other voltammetric methods for ACL determination that were described in the literature. To verify the usefulness of the method, the determination of ACL in real samples with different matrices was performed. Measurements were carried out successfully in tablets containing ACL, urine, and plasma. The recoveries were in the range of 98% to 105%, suggesting a good accuracy of the method. On the basis of the conducted research, it can be concluded that the developed voltammetric method for ACL determination might be a useful tool in routine ACL measurements.

## Figures and Tables

**Table 1:** Comparison of LOD and LOQ values for ACL determination using different voltammetric methods.

Type of Working Electrode	LOD, nM	LOQ, nM	Reference
OH-MWCNT/CPE	246	Not specified	[10]
CMCPE	19.3	Not specified	[11]
EPG	Not specified	70	[12]
MCPGE	2.6	Not specified	[13]
eCNF/CNT/NiCo-GCE	0.7	2.1	This work

OH-MWCNT/CPE—OH-functionalized multiwalled carbon nanotube carbon paste electrode. CMCPE—surfactant chemically modified carbon paste electrode. EPG—edge plane pyrolytic graphite electrode. MCPGE—multiwalled carbon nanotubes-modified pencil graphite electrode.

**Table 2:** Results of the determination of ACL in pharmaceutical product, urine and plasma samples.

Sample	ACL Added, mg	ACL Found ± s, mg, (Recovery, %)
Tablet ^a^	0	110 ± 4
	100	220 ± 2 (103)
	200	325 ± 7 (103)
Sample	ACL added, µM	ACL found ± s, µM, (recovery, %)
Urine diluted 20×	0	ND
	0.2	0.19 ± 0.01 (98)
	0.4	0.39 ± 0.02 (99)
Urine diluted 10×	0	ND
	0.2	0.21 ± 0.01 (105)
	0.4	0.42 ± 0.03 (105)
Plasma diluted 33×	0	ND
	0.2	0.19 ± 0.02 (98)
	0.4	0.42 ± 0.03 (105)
Plasma diluted 20×	0	ND
	0.3	0.29 ± 0.02 (98)
	0.6	0.63 ± 0.05 (104)

^a^ Producer declares 100 mg per tablet. ND—not detected.

## Data Availability

The data presented in this study are available on request from the corresponding author.
